# Comparison of Two Toric IOL Calculation Methods

**DOI:** 10.1155/2018/2840246

**Published:** 2018-01-10

**Authors:** C. Kern, K. Kortüm, M. Müller, A. Kampik, S. Priglinger, W. J. Mayer

**Affiliations:** Department of Ophthalmology, Ludwig-Maximilians-University, Munich, Germany

## Abstract

**Purpose:**

To compare two calculators for toric intraocular lens (IOL) calculation and to evaluate the prediction of refractive outcome.

**Methods:**

Sixty-four eyes of forty-five patients underwent cataract surgery followed by implantation of a toric intraocular lens (Zeiss Torbi 709 M) calculated by a standard industry calculator using front keratometry values. Prediction error, median absolute error, and refractive astigmatism error were evaluated for the standard calculator. The predicted postoperative refraction and toric lens power values were evaluated and compared after postoperative recalculation using the Barrett calculator.

**Results:**

We observed a significant undercorrection in the spherical equivalent (0.19 D) by using a standard calculator (*p* ≤ 0.05). According to the Baylor nomogram and the refractive influence of posterior corneal astigmatism (PCA), undercorrection of the cylinder was lower for patients with WTR astigmatism, because of the tendency of overcorrection. An advantage of less residual postoperative SE, sphere, and cylinder for the Barrett calculator was observed when retrospectively comparing the calculated predicted postoperative refraction between calculators (*p* ≤ 0.01).

**Conclusion:**

Consideration of only corneal front keratometric values for toric lens calculation may lead to postoperative undercorrection of astigmatism. The prediction of postoperative refractive outcome can be improved by using appropriate methods of adjustment in order to take PCA into account.

## 1. Introduction

During the last few years, the demand of patients undergoing cataract surgery for age-related cataract has increased. Not only cataract extraction itself but also satisfactory postoperative refractive results are mandatory for good visual function. Moreover, artificial lens material has improved over time and complication rates have decreased [1, 2]. Therefore, the expectations of patients undergoing cataract surgery are almost the same as those for refractive surgery, although preoperative astigmatism of more than 1.5 dioptres (D) is present in 20% of all cataract patients [3]. Residual postoperative astigmatism is an important cause for the lack of achievement of emmetropia after successful cataract surgery [4].

Toric intraocular lenses are a major advance in reducing corneal astigmatism through cataract surgery [5]. In contrast, more than one-third of patients do not reach target refraction after toric lens implantation [6]. A higher error in refractive astigmatism (ERA) has been shown to be obtained by ignoring the posterior corneal astigmatism (PCA) in toric lens calculation [7]. The most commonly used biometers (manual and IOLMaster 500) rely purely on measurements of the anterior curvature when measuring corneal astigmatism. This may lead to poor prediction of the total corneal astigmatism (TCA). By using nomograms such as the Baylor nomogram or the Barrett calculator, PCA can be predicted dependent on the power and axis of the ACA [8, 9]. The lowest predicted residual astigmatism has been reached by using the Barrett toric calculator [10]. Adjustment of standard industry-based calculators by a new regression formula to calculate the TCA can improve the error in predicted postoperative astigmatism [11].

The influence of the PCA is different for eyes with an anterior with-the rule (WTR) or against-the-rule (ATR) astigmatism. The TCA is overestimated in WTR eyes (0.5 to 0.6 D) and underestimated in ATR eyes (0.2 to 0.3 D) when planning toric lens implantation without considering the PCA [12].

The objective of our study was to evaluate a standard industry calculator by using IOLMaster corneal front keratometry values followed by recalculation using the Barrett calculator. Therefore, we measured postoperative refraction and postoperative refractive error for the standard calculator and compared the predicted target refraction and the suggested intraocular toric lens power between the calculators. By adapting the refractive index to 1.3375, the anterior corneal astigmatism (ACA) could be calculated and used by the industry-based calculator. The Barrett calculator estimated TCA [10].

## 2. Material and Methods

Sixty-four eyes of forty-five patients were included in a retrospective case series at the University Eye Hospital in Munich, Germany. Ethical aspects were considered and the guidelines of Helsinki Declaration were followed. The study has been approved by the Institutional Review Board of the University Eye Hospital in Munich. Patients with age-related cataract and preoperative regular astigmatism of more than 1.0 D by topographic Scheimpflug analysis (OCULUS Pentacam, Wetzlar, Germany) were included. Exclusion criteria were pseudoexfoliation syndrome, corneal pathologies, irregular astigmatism, glaucoma, prior vitreoretinal surgery, or any kind of maculopathies. Furthermore, patients with a documented realignment of the toric intraocular lens during follow-up were also excluded for study purposes. For optical biometry, IOLMaster 500 (Zeiss Meditec, Oberkochen, Germany) was used and preoperative Scheimpflug measurement for total corneal astigmatism assessment was carried out in all patients. These patients underwent coaxial microincision high fluidic cataract surgery (CoMICS) and phacoemulsification (Oertli OS3 device, Oertli, Switzerland) by an experienced surgeon (A.K.) who carried out a clear corneal incision (CCI) at 90° with an estimated surgeon-specific surgically induced astigmatism of ≤0.5 D followed by implantation of a toric intraocular lens (Zeiss Torbi 709 M) calculated by a standard industry-based calculator (ZCalc, Zeiss Meditec, Oberkochen, Germany). We avoided the performance of CCI from the temporal position in ATR astigmatism patients, because all surgeries were performed by an experienced cataract and vitreoretinal surgeon routinely employing a position at 90° as a surgical approach. Moreover, limitation of the specific surgically induced astigmatism to one axis simplifies the evaluation of postoperative astigmatism. The toric IOL was intraoperatively aligned by using the Callisto Eye (Zeiss Meditec, Oberkochen, Germany) digital tracking system with regard to a reference image assessed with the IOLMaster 500. To evaluate the standard calculator's clinical outcome, firstly, the prediction error (PE) was calculated as followed: “actual refraction to predicted refraction.” After zeroing out the arithmetic mean error, the median absolute error (medAE) was determined following an established method described by Hoffer et al. and later discussed as state of the art by Wang et al. [13, 14]. Considering the preoperative calculations of the standard and the Barrett calculator, the difference of predicted postoperative refraction values and the lens suggestion between calculators were analysed and compared. We used the same IOLMaster 500 values from the preoperative measurement for both the standard calculator and the Barrett calculator. Postoperative refraction was confirmed by using an autorefractometer (Nidek AR-1, Japan) at least six weeks after surgery. Since October 2012, all patient contacts in our clinic have been recorded digitally in a custom-made electronic health record (EHR) [15]. All findings have been exported in a data warehouse called the Smart Eye Database (SMEYEDAT) including clinical data (e.g., diagnoses, visit date, and visual acuity) and diagnostic devices (e.g., IOLMaster and Pentacam). A SMEYEDAT query for all included patients was performed to obtain refraction parameters, IOLMaster 500 and Pentacam values. The data were exported in an Excel spreadsheet for further statistical analysis. Study eyes were divided into groups according to the steep astigmatic axis: with-the-rule (60–120°), against-the-rule (0–30° or 150–180°), and oblique (31–59° and 121–149°). For all statistical analysis, IBM SPSS Statistics Version 24 was used. Wilcoxon statistical testing was performed to measure the statistical difference between two parameters and for subgroup analysis. *P* values of 0.05 or less were considered statistically significant.

Consideration of the postoperative astigmatism and axis separately does not allow the determination as to whether over- or undercorrection has occurred. Therefore, the power (D) and axis (degree) of postoperative astigmatism have to be described based on one variable. This was achieved by performing vector analysis following the Alpins method [16]. The preoperative corneal astigmatism was defined as the target-induced astigmatism (TIA) and describes the astigmatism change intended to be induced through surgery. Full correction of astigmatism was intended in all cases. Owing to the lack of postoperative keratometry measurements and verification of lens alignment, the residual postoperative astigmatism out of manifest refraction was defined as the difference vector (DV), which refers to the postoperative refractive astigmatism error. These two vectors were used to calculate and validate total surgically induced astigmatism (TSIA) by means of the assort vector calculator (http://www.assort.com/assort-vector-calculator-0; accessed 10.12.2016). TSIA mirrors the entire amount of astigmatism change through surgery, including both the SIA by corneal incisions and the amount induced by toric cylinder power. Afterwards, calculation of mean vectors from the TIA, TSIA, and DV was performed by the summated vectorial mean method according to Alpins and Goggin [17]. These three mean vectors were then used to calculate the various astigmatic parameters and indices that can be employed to determine the overall success of astigmatism correction (index of success, flattening index, and flattening effect), possible over- or undercorrection (correction index), and the misalignment of treatment (angle of error) [16].

In detail, the index of success is calculated by dividing the DV by the TIA and is preferably “0.” A DV of “0” would imply no postoperative residual astigmatism. The flattening effect (FE) describes the amount of astigmatism reduction by the TSIA at the intended axis (axis of TIA) and is used to calculate the flattening index (FI). The FI itself is calculated by dividing the FE by the TIA and is “1” in the ideal case. This would mean that the whole amount of TSIA was induced at the axis of TIA. The correction index (CI) is calculated by dividing the TSIA by TIA and is “1” if the complete TIA has been corrected. If this value is above 1, then overcorrection has occurred, whereas if it is below 1, then undercorrection has occurred. The angle of error (AoE) compares the misalignment of the axis of TIA and TSIA.

## 3. Results

Mean age of all patients was 66.0 ± 16.7 years. Of the 64 observed eyes, 35 were male and 29 female. In 19 patients, both eyes were included. The preoperative uncorrected mean visual acuity was 0.50 logMAR and the target refraction, as defined by the surgeon in our EHR, was −0.36 ± 0.68 D in the sphere with full correction of the astigmatism. A summary of the demographic and clinical data including pre- and postoperative refraction is shown in Table 1. The improvement of postoperative uncorrected visual acuity at 0.20 logMAR was statistically significant compared with preoperative values (*p* < 0.01).

When comparing the predicted postoperative refraction by the standard calculator with the manifest postoperative refraction, we used the prediction error as well as the median absolute error for SE and cylinder. Considering the SE, we defined overcorrection as manifest refraction more myopic than the predicted refraction (the chosen SE lens power was too strong) and undercorrection as manifest refraction more hyperopic than the predicted refraction (the chosen SE lens power was too weak). By using a standard calculator, we reached a statistically significant prediction error of 0.19 D in mean SE (*p* = 0.047). Postoperative undercorrection was attained. We performed the same analysis for subgroups of eyes with preoperative WTR (*n* = 47) and ATR (*n* = 12) and oblique astigmatism (*n* = 5). On the basis of the small numbers, patients with oblique astigmatism were excluded from the subgroup analysis. In WTR eyes, the prediction error was 0.20 D and again undercorrection was attained but statistical significance was absent. The prediction error was 0.20 D for ATR eyes and no significant difference between predicted and postoperative refraction was found.

When comparing the predicted postoperative cylinder, we observed a statistically significant prediction error of −0.60 D when considering all eyes (*p* < 0.01). A negative prediction error cylinder is defined as undercorrection of the preoperative cylinder. Positive results are compatible with overcorrection. In WTR eyes, the PE was −0.52 (*p* < 0.01) as against ATR eyes in which it was −1.00 (*p* < 0.01). Analogical results for all patients and the subgroups were contained when considering the median absolute errors. A summary of the prediction errors and the median absolute errors for SE and cylinder when using a standard industry calculator can be found in Table 2.

A direct comparison between the standard industry calculator and the Barrett calculator with same optical biometry values and target refraction revealed a statistically significant difference regarding predicted postoperative refraction. In SE and sphere, the Barrett calculator predicted less overcorrection compared with target refraction (*p* < 0.01). The Barrett calculator predicted a lower residual cylinder than the standard calculator expected. For the predicted postoperative refraction of the various calculators, we performed a subgroup analysis for patients with WTR and ATR astigmatism (Table 3).

For patients with WTR astigmatism, the standard calculator predicted a more myopic postoperative refraction in predicted SE (−0.70 D versus −0.35 D; *p* < 0.01) and sphere (−0.52 D versus −0.25 D; *p* < 0.01) and higher residual cylinder (−0.37 D versus −0.19 D; *p* < 0.01). In addition, for ATR astigmatism, the standard calculator again predicted a more myopic postoperative refraction in SE and sphere. Both results were statistically significant (*p* < 0.01). The difference of the predicted residual cylinder did not reach the required level of significance. With regard to the suggested toric lens power between calculators, the Barrett calculator suggested a lower power of sphere (17.55 D versus 17.28 D; *p* = 0.01) and toric cylinder (3.22 D versus 3.00 D; *p* = 0.26) in all patients. In both subgroups, these results were repeated and the Barett calculator suggested a lower sphere and cylinder power. Only the difference of suggested cylinder power in the ATR astigmatism group statistical significance was absent. Both calculators predicted the same lens orientation axis for the toric IOL (87.4° versus 87.9°, *p* = 0.49). A summary of the predicted lens power and orientation is given in Table 4. During a comparison of the orientation of the toric cylinder power axis, the standard deviation must be considered. The lens orientation in WTR eyes was 89.53° versus 89.87° as against 75.00° versus 75.17° in ATR eyes. With regard to the standard deviation in WTR (10.47° and 12.63°) and ATR (75.49° and 78.86°) eyes, a higher dispersion around approximately 75° was shown in ATR eyes suggesting a lens orientation approximately around 0° and 150°.

**Figure 1:**
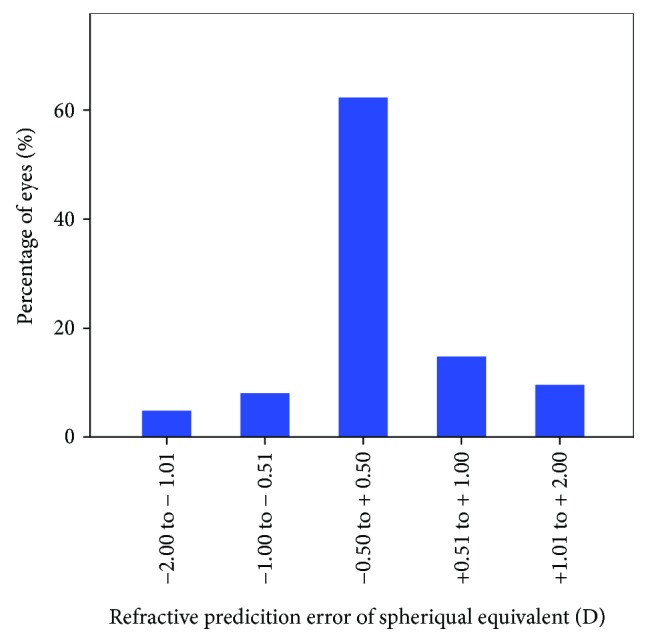
Percentage of eyes within a certain range of refractive prediction error (spherical equivalent) when using a standard industry-based calculator.

**Figure 2:**
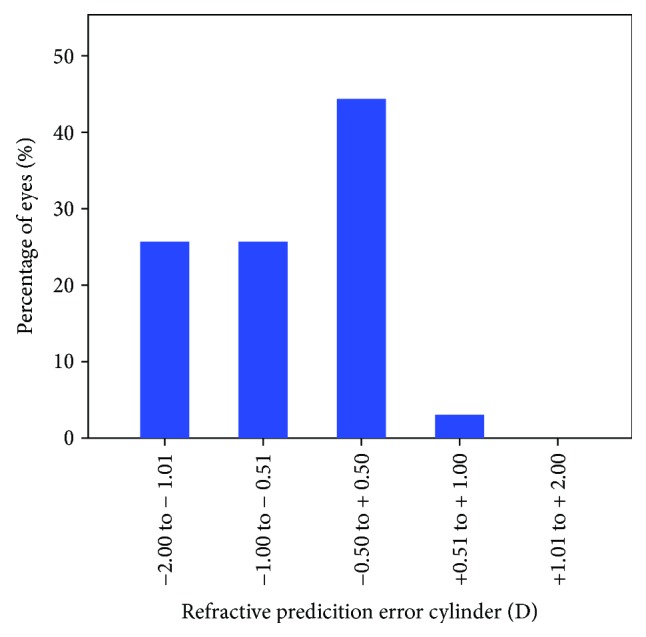
Percentage of eyes within a certain range of refractive predication error (cylinder) when using a standard industry-based calculator.

Almost 60% of the patients had a prediction error of SE within the limits of ±0.5 D. The mean postoperative residual cylinder was −1.0 D with a standard calculator. For 44% of patients, the prediction error of the residual cylinder was within ±0.5 D (Figures 1 and 2).

For vector analysis of astigmatism when using a standard industry calculator, the mean target-induced astigmatism was calculated as 1.88 D with an axis of 85°. Over the residual postoperative astigmatism, the difference vector was calculated to be 1.38 D at 86° generating a total surgically induced astigmatism of 0.78 at 99°. A summary and the results including the subgroups can be found in Table 5.

Multiple variables were calculated based on these three basic vectors. The correction index was 0.97 for all eyes. For the subgroups, the CI was 1.00 in WTR and 0.69 in ATR eyes. The angle of error differed between 7.41° in WTR and 9.11° for ATR eyes, being 2.21 for all eyes. The index of success was 0.56 in all eyes within a range of 0.48 for WTR and 0.61 for ATR eyes. In all eyes, we calculated the flattening index (FI) from the flattening effect (FE) as 0.76. Mean FE was 1.91 in all eyes: 2.12 for WTR and 1.44 for ATR eyes.  Table 6 gives an overview of all calculated variables from the three mean vectors including the subgroups of WTR and ATR eyes.

**Figure 3:**
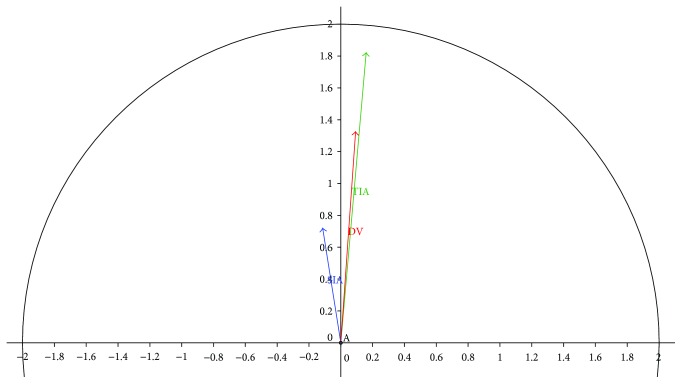
Visualization of the mean vectors from the vector analysis: target-induced astigmatism (1.88 D; 85°), total surgically induced astigmatism (0.78 D; 99°), and difference vector (1.38 D; 86°).

**Figure 4:**
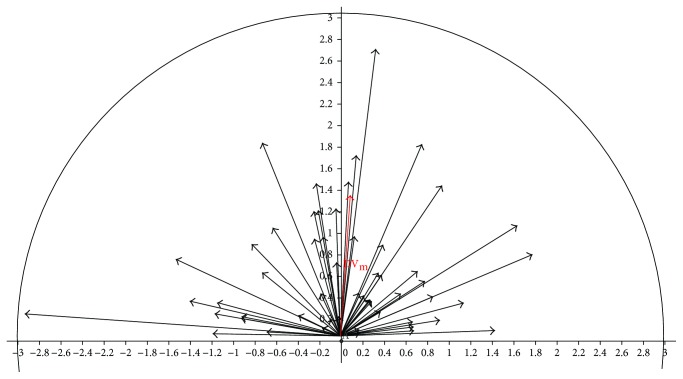
Visualization of all difference vectors from the vector analysis (*n* = 64); DV_m_ = mean difference vector.

The mean vectors (TIA, DV, and TSIA) for all eyes are visualized in Figure 3, whereas Figure 4 shows the distribution of the DV in order to visualize the deviation of residual postoperative astigmatism.

## 4. Discussion

By using an industry-based calculator for toric lens implantation, we reached statistically significant postoperative undercorrection for SE (0.19 D; *p* = 0.047). By using SE, the prediction error was within a range between 0.08 and 0.20 D for all subgroups (including oblique astigmatism). Considering the prediction error of the cylinder, we observed a −0.60 D (*p* < 0.01) deviation from the predicted residual cylinder in all eyes which equals an undercorrection of the preoperative cylinder (Table 2).

Previous clinical studies have reported that the Barrett toric calculator has an advantage over established calculators such as other industry-based calculators or the Holladay toric calculator. In an earlier study with the Barrett calculator, 76% and 93% of eyes were within 0.50 and 0.75 D residual astigmatism [18]. In our patients, only 31% and 63% had a residual cylinder less than 0.50 and 1.00 D following use of an industry-based calculator; our data allow the assumption of an advantage of the Barrett calculator in toric lens calculation.

The error of predicted residual astigmatism (difference between predicted and actual postoperative cylinder) was between 0.01 D and 0.16 D in another study using the Barrett calculator [10]. In our study group, the astigmatic prediction error was between −0.52 D and −1.00 D, a result that suggests the superiority of the Barrett calculator over a standard calculator when only corneal front *K* values are used. To evaluate postoperative over- or undercorrection of astigmatism more precisely, the axial shift of postoperative astigmatism must be included and not only astigmatic power. This was achieved by vector analysis according to the Alpins method [16].

The limitation of our study was that the postoperative refraction including astigmatism was determined by manifest refraction, whereas keratometry values were missing from the follow-up examinations. Therefore, we were unable to separate the influence of surgically and lens-induced changes in astigmatism. Owing to the retrospective study design, it was not possible to include the actual postoperative orientation of the toric lens in analysis. As stated in Materials and Method, patients with a postoperative realignment of the toric intraocular lens during the follow-up period were excluded from this study. As surgically induced astigmatism was always considered with 0.5 D at 90° in the calculation, we can nevertheless make conclusion concerning the accuracy of the calculator. Moreover, the minimum threshold for a refractive change in objective refraction measurement is a change of ±0.25 D in the actual refraction of the patient's eyes [19]. This may lead to inaccuracy when comparing pre- and postoperative refraction. The main focus in this study was to compare the accuracy of various calculators in the calculation of predictive outcome for toric lens calculation in a retrospective case series and to evaluate the postoperative results when using a standard calculator.

Regarding only the SE, WTR and ATR eyes tended to an undercorrection of 0.20 D. These results only consider the spherical equivalent and may be influenced by prediction error in the sphere. Koch et al. have stated the role of PCA in toric lens calculation; in eyes with WTR astigmatism, the total corneal astigmatism is overestimated by 0.5 to 0.6 D, whereas for ATR astigmatism, it is underestimated by 0.2 to 0.3 D [12]. Hereby, eyes with WTR or ATR astigmatism tend to over- or undercorrection without regarding the Baylor nomogram [12, 20, 21]. According to this supposition, WTR eyes should be overcorrected and ATR eyes undercorrected. In our patients, only considering the PE, we reached less undercorrection in WTR eyes compared to ATR eyes (−0.52 D versus −1.00 D). The difference between the groups was statistically significant (*p* = 0.044). Taking into account that all patients in our study were undercorrected when using a standard calculator (PE = −0.60 D; *p* < 0.01), our WTR patients were indeed “overcorrected” and ATR patients were, respectively, “undercorrected.” To evaluate the possible over- or undercorrection of astigmatism more precisely, the values from the vector analysis must be taken into account.

The Barrett calculator results in lower postoperative residual astigmatism and, since an industry-based calculator is optimized for the company-owned toric lens, we reevaluated the two calculators retrospectively. We compared predicted postoperative refraction and the suggested lens power between the calculators for the same target refraction. This was −0.36 D in the sphere and 0.00 D in cylinder in all eyes, which is equivalent to an SE of −0.36 D. The predicted postoperative refraction of the Barrett calculator with the same optical biometry values was predicted closer to the defined target refraction of the surgeon in the SE, sphere, and cylinder (*p* < 0.01). Moreover, the predicted postoperative residual cylinder was less by using the Barrett calculator (−0.21 D versus −0.37 D; *p* < 0.01). The same findings were approved for all astigmatism subgroups (Table 3). By using the Barrett calculator, the predicted postoperative refraction was closer to target refraction for the spherical equivalent, sphere, and astigmatism than by using a standard calculator. By adjusting the standard calculators with new nomograms considering the TCA of the cornea, the error of prediction can be reduced to similar values to those that the Barrett calculator delivers [11].

For all patients, the Barrett calculator suggests significant lower lens power for the sphere but not for toric cylinder power. For astigmatism subgroup analysis, the Barrett calculator suggests less spherical toric lens power compared with an industry-based calculator for WTR and ATR eyes. Only in WTR eyes, this difference was significant. In contrast, in ATR eyes, the Barrett calculator suggests a stronger spherical component (Table 4). These results can be explained based on the expected overcorrection in WTR astigmatism and undercorrection in ATR astigmatism, as is often described in the literature [21, 22]. The Barrett calculator weakens lens power in WTR astigmatism to avoid overcorrection and strengthens it in ATR astigmatism to avoid the expected undercorrection. This correlation only missed the significance for cylinder power in ATR eyes probably due to the low number of patients in this subgroup. The distribution on WTR and ATR eyes in this study was similar to the literature (WTR: 73% versus 61%; ATR: 18% versus 20%) [23]. After recalculation by using the Barrett calculator, the undercorrection of spherical equivalent in all eyes might have been prevented if the suggested lens by this calculator would have been used.

To determine the effectiveness of the astigmatic correction while using a standard calculator, a vector analysis according to the Alpins group was performed. Alpins astigmatism analysis is based on three fundamental vectors: the TIA, TSIA, and DV. These three vectors are then used to calculate the various astigmatic parameters and indices that can be used to determine the overall success of astigmatism correction (index of success and flattening index), possible over- or undercorrection (correction index), and misalignment of treatment (angle of error) [16, 17]. A precise explanation of the various vector analysis indices can be found in the Materials and Methods. The DV, which displays residual astigmatism, is higher at 1.38 than that obtained in other studies examining outcomes after toric lens implantation. A DV between 0.3 and 0.87 was reached in the literature for toric lens implantation [24, 25]. The correction index in our collective was below 1 (0.97), which means that we reached an undercorrection of corneal astigmatism by using a standard calculator. Similar studies have shown a correction index between 0.95 and 1.09 [24, 26, 27]. In WTR eyes, the correction index was 1.00 and, in ATR eyes, it was 0.69. Thus, we reached a mean full correction in WTR eyes and a strong undercorrection of astigmatism in ATR eyes. As stated previously, according to Koch et al., astigmatism in WTR eyes is mostly overrated, whereas it is underrated in ATR eyes without consideration of the TCA [12]. Our results are nevertheless compatible with the hypothesis of Koch as we reached a stronger undercorrection in ATR eyes (CI = 0.69) than in WTR eyes (CI = 1.00), whereas we achieved slight undercorrection in all eyes (CI = 0.97) when using the corneal front *K* values in the standard calculator only.

The angle of error in the mean showed a difference of 2.21° between the axis of TSIA compared with the axis of TIA. This result is similar to that of another study examining postoperative results of a trifocal toric lens [28]. We attained an index of success (DV/TIA) of 0.56, a value that should be 0 in the ideal case. In other studies involving the performance of vector analysis after toric lens implantation, the index of success was below 0.5 [25]. Both DV and TIA were measured via objective refraction. The FE was 1.91 and showed the amount of astigmatism reduction achieved by the effective proportion of the SIA at the intended meridian. This is mainly used to calculate the flattening index (FI). The FI should preferably be 1.0 and was 0.76 in our patients. The FI was calculated by dividing the FE by the TIA and values below 1.0 can be interpreted as undercorrection of the postoperative cylinder. Again, undercorrection was higher in ATR than in WTR eyes confirming the results obtained by calculating the correction index.

The inaccuracy of DV and the index of success rate can be explained based on the higher scattering that occurs by using the objective refraction measurement than by using the optical biometry to determine postoperative refraction and on the overall retrospective study design.

In summary, we attained postoperative undercorrection by using a standard calculator in the spherical equivalent (PE = 0.19 D) and postoperative residual astigmatism (CI< 1). For postoperative astigmatism, WTR eyes were less undercorrected (CI = 1.00) than ATR eyes (0.69), a finding that can be explained with the Baylor nomogram [12]. A comparison of the two calculators predicts that target refraction in the sphere and cylinder is closer to the target refraction as determined by the surgeon and that the expected prediction error is less when reevaluating biometry values with the Barrett calculator. These results confirm the advantages of the Barrett calculator over corneal front-based toric lens calculators. The importance of PCA in predicting postoperative refraction after toric lens implantation is highlighted by these results. A prospective study setting, observing the postoperative refractive outcome between the calculators and including the postoperative lens orientation would be helpful to understand and optimize refractive outcomes in toric lens calculation.

## Figures and Tables

**Table 1:** Summary of demographic and clinical data (by eye) of data set.

Patients (*n*)	45
Eyes (*n*)	64
Sex (male : female) (*n*)	35 : 29
Age (years)	66.0 ± 16.7 (37 to 92)
Axial length (mm)	24.31 ± 2.09 (20.84 to 30.94)
Target refraction (D)	−0.36 ± 0.68

*Preoperative data*	
Spherical equivalent (OR) (D)	−1.90 ± 5.15 (−16.00 to 8.60)
Sphere (OR) (D)	−0.81 ± 5.19 (−14.75 to 10.75)
Astigmatism (OR) (D)	−2.32 ± 1.39 (−6.00 to −0.25)
Axis (OR) (°)	95.0 ± 69.0 (0.0 to 180.0)
Mean visual acuity (logMAR)	0.50 ± 0.70

*Postoperative data*	
Spherical equivalent (OR) (D)	−0.48 ± 1.09 (−2.75 to 2.00)
Sphere (OR) (D)	0.00 ± 1.12 (−2.50 to 2.75)
Astigmatism (OR) (D)	−0.97 ± 0.65 (−3.00 to 0.00)
Axis (OR) (°)	95.7 ± 57.2 (2.0 to 180.0)
Mean visual acuity (logMAR)	0.20 ± 0.60

**Table 2:** Refractive prediction error and median absolute error for spherical equivalent and cylinder when using a standard industry calculator.

		Prediction error	Median absolute error	*p* value
Spherical equivalent	All (*n* = 64)	0.19 ± 0.82	−0.04 ± 0.82	0.05
	WTR (*n* = 47)	0.20 ± 0.81	0.00 ± 0.81	0.18
	ATR (*n* = 12)	0.20 ± 1.00	0.01 ± 1.00	0.16

Cylinder	All (*n* = 64)	−0.60 ± 0.67	0.05 ± 0.67	≤0.01
	WTR (*n* = 47)	−0.52 ± 0.64	0.08 ± 0.64	≤0.01
	ATR (*n* = 12)	−1.00 ± 0.78	−0.37 ± 0.78	≤0.01

**Table 3:** Comparison of the predicted postoperative refraction estimated by the standard and the Barrett calculator. Subgroup analysis for with-the-rule (WTR) and against-the-rule (ATR) astigmatism.

Subgroup	Calculator	Mean SE (D)	Mean sphere (D)	Mean cylinder (D)
All (*n* = 64)	Standard	−0.67 ± 0.77	−0.49 ± 0.81	−0.37 ± 0.29
	Barrett	−0.38 ± 0.73	−0.10 ± 1.10	−0.21 ± 0.20
	*p* value	<0.01	<0.01	<0.01

WTR (*n* = 47)	Standard	−0.70 ± 0.80	−0.52 ± 0.84	−0.37 ± 0.27
	Barrett	−0.35 ± 0.73	−0.25 ± 0.71	−0.19 ± 0.18
	*p* value	<0.01	<0.01	<0.01

ATR (*n* = 12)	Standard	−0.46 ± 0.44	−0.24 ± 0.41	−0.43 ± 0.37
	Barrett	−0.31 ± 0.47	0.75 ± 1.82	−0.28 ± 0.26
	*p* value	<0.01	<0.01	0.14

**Table 4:** Comparison of the suggested lens power of sphere and cylinder including toric cylinder orientation by the standard and the Barrett calculator. Subgroup analysis for with-the-rule (WTR) and against-the-rule (ATR) astigmatism.

Subgroup	Calculator	Mean spherical power (D)	Mean cylinder power (D)	Lens orientation (°) ^∗^
All (*n* = 64)	Standard	17.55 ± 5.95	3.22 ± 2.07	87.42 ± 35.76
	Barrett	17.28 ± 6.83	3.00 ± 1.54	87.92 ± 38.44
	*p* value	0.01	0.26	0.49

WTR (*n* = 47)	Standard	17.67 ± 5.85	2.98 ± 1.32	89.53 ± 10.47
	Barrett	17.02 ± 7.01	2.75 ± 1.50	89.87 ± 12.63
	*p* value	0.05	< 0.01	0.628

ATR (*n* = 12)	Standard	18.04 ± 5.83	4.71 ± 3.70	75.00 ± 75.49
	Barrett	19.17 ± 5.43	4.31 ± 1.36	75.17 ± 78.86
	*p* value	0.08	0.50	0.84

^∗^The standard deviation must be considered when interpreting the toric cylinder orientation. For WTR and ATR, the mean values are almost the same. With regard to the standard deviation, most of the values indeed lie around 90° for WTR, whereas for ATR, only the mean is approximately 75° but the actual values are around 0° or 150°.

**Table 5:** Vector analysis for target-induced astigmatism (TIA), total surgically induced astigmatism (TSIA), and the difference vector (DV) including subgroup analysis for with-the-rule (WTR), against-the-rule (ATR), and oblique preoperative astigmatism.

	Target-induced astigmatism	Difference vector	Total surgically induced astigmatism
All patients (*n* = 64)	1.88; 85°	1.38; 86°	0.78; 99°

WTR (*n* = 47)	2.44; 91°	0.90; 76°	2.32; 98°

ATR (*n* = 12)	2.48; 88°	1.29; 99°	1.69; 79°

TIA refers to the preoperative astigmatism of the patient's eye in manifest refraction. TSIA refers to the amount of corrected astigmatism in surgery (induced by corneal incisions and toric IOL). DV refers to the residual postoperative astigmatism of the patient's eye.

**Table 6:** Calculation of typical variables in vector analysis.

	Correction index	Angle of error (°)	Index of success	Flattening index
All patients (*n* = 64)	0.97	2.21	0.56	0.76

WTR (*n* = 19)	1.00	7.41	0.48	0.84

ATR (*n* = 16)	0.69	−9.11	0.61	0.52

Correction index: >1.0 = overcorrection; <1.0 = undercorrection; Angle of error: between the axis of TIA and TSIA (“+” = counterclockwise; “−”=clockwise). Index of success is obtained by dividing DV by TIA. Preferably = 0. Flattening index is obtained by dividing FE by TIA. Preferably = 1.0.
